# Image-Based Approach Applied to Load Torque Estimation in Three-Phase Induction Motors

**DOI:** 10.3390/s24082614

**Published:** 2024-04-19

**Authors:** Cleber Gustavo Dias, Jhone Fontenele

**Affiliations:** Informatics and Knowledge Management Graduate Program, Nove de Julho University—UNINOVE, Sao Paulo 01525-000, Brazil; fontenele.jhone@uni9.edu.br

**Keywords:** torque estimation, image-based condition monitoring, convolutional neural networks

## Abstract

This paper presents a novel method for load torque estimation in three-phase induction motors using air gap flux measurement and the conversion of this type of time-domain signal into grayscale images for further processing as inputs for an inception-type convolutional neural network. The magnetic flux was measured employing a Hall effect sensor installed inside the machine, near the stator slots, and above the stator windings. In this case, the sensor was able to measure a resultant magnetic flux density, having both rotor and stator magnetic flux contributions. The present methodology does not require motor parameters for torque prediction. The proposed approach successfully estimated load torque using three optimizers across almost the entire motor load operational range, spanning from 1.5% to 93.9% of the rated load. Four model configurations achieved a mean absolute percentage error (MAPE) less than or equal to 3.7%. Specifically, two models for a 40 × 50 pixel image achieved MAPE of 3.7% and 3%, one model for a 40 × 25 pixel image achieved a MAPE of 3.5%, and one model for a 50 × 80 pixel image achieved a MAPE of 3.3%. This research has been experimentally validated with a 7.5 kW squirrel cage induction machine.

## 1. Introduction

Rotating electrical machines are the main industrial assets responsible for energy conversion in productive segments, in a variety of applications. In this sense, three-phase squirrel cage induction motors (TIMs) have been the most employed machines in the last decades in the industrial environment, being responsible for operating in different types of drives [1]. Despite the widespread use of TIMs, their measured or estimated torque and efficiency are important information for energy diagnosis purposes and to match the motor load [2]. In addition, as cited by [3,4], knowing the torque value helps professionals and engineers better understand potential faults or malfunctions in the machine [5].

The monitoring of load torque can be directly assessed through a torque sensor, often referred to as a torque meter. This technique, known as direct torque measurement, is also addressed by some technical approaches [6], but using a torque meter has significant drawbacks due to its difficulty in installation on the shaft, between the load and the TIM [7]. Nonetheless, the mechanical installation between rotating components renders this approach unviable in various scenarios, particularly within critical systems [8]. The precise alignment of torque meters with the motor shaft is imperative, as any misalignment can curtail the operational lifespan of these instruments, resulting in protracted and costly installation procedures. Given these constraints, numerous ongoing studies are focused on estimating load torque indirectly [9], obviating the need for mechanical installation or coupling between the TIM and the mechanical system.

During the past decades, many researchers have dedicated themselves to investigating new techniques and approaches for estimating load torque in induction motors [2,3,4,5,6,7,8].

Recently, new research has led to the use of airgap and stray flux measurements for the condition monitoring of motors, including fault diagnosis [10,11,12] and load torque estimation [4].

In this sense, the authors [8] proposed an alternative approach by indirectly measuring the torque using parameters such as three-phase currents, line voltage, and stator resistance. This approach involves calculating the electromagnetic air-gap torque (AGT) of the TIM. The approach showcases notable benefits, demonstrating estimation errors below 2% for load levels spanning 20% to 110% of the torque nominal range. Nevertheless, as the load descends below 20% of the motor’s nominal load capacity, the model’s estimation capability weakens, resulting in increased errors of up to 5%.

The AGT is one of the most known techniques [13]; however, in this approach, it is necessary to apply several measurements, such as the voltages and currents of the TIM.

Similarly, the work by [14] also addresses the AGT estimation based on root mean square (RMS) values of variables such as current, voltage, and power. This work suggests presenting a method relying solely on the RMS values. However, a limitation of this method is in the intricacies of modeling and quantifying the losses within the motor, as highlighted by the authors [14]. These losses pose a persistent challenge due to the complexity involved in their calculation.

The research conducted by [15] underscores the significance of diagnosing efficiency in rotating machines by assessing load torque. It introduces a method for estimating torque by using electric current and magnetic flux from the motor stator. This method proposes a non-invasive technique for gauging the stator’s magnetic flux within the TIM, achieved through the utilization of an external copper coil. This coil replicates the air gap flux during motor operation. By incorporating the AGT method and these parameters in the TIM model, the study facilitates estimations of electromagnetic torque. However, relying solely on the external copper coil for estimating electromagnetic torque reveals imprecision, which is particularly evident with errors exceeding 10% at lower loads, posing an identifiable limitation. Nonetheless, when combining the magnetic flux from the coil with the stator current, the errors diminish. Specifically, the combined approach reduces errors to 8.5% under no-load conditions and drops further to below 4.1% at full load, showcasing substantial improvement in accuracy.

Recently, many approaches have been put forward in the literature to suggest different alternatives to asses the TIM’s patterns based on its produced data [16]. Groundbreaking results have emerged, demonstrating the various benefits of applying a data-driven approach to motors, as proposed by [17], which is based on simulated and real data for estimating the speed and torque of a TIM through a computational model, considering a convolutional encoder-decoder neural network architecture. According to the authors [17], the data-driven approach to estimating speed and torque from measured currents and voltages of a TIM has allowed the bridging of simulated data from a physical model to the real world, using convolutional neural network techniques to learn the underlying dynamic model of the TIM.

Currently, artificial neural network (ANN) models based on data, with capabilities similar to or even better than those based on mathematical models of TIM, have further encouraged researchers to seek meaningful insights into the dynamic behavior of the motor through data. For example, the study conducted by [18], which employs an ANN model considering a linear regression (LR) algorithm with supervised learning, estimates load torque while incorporating signal processing techniques.

In the continuous pursuit of enhancing the performance of electric motors, recent studies demonstrate how convolutional neural networks can optimize topology (TO—topology optimization) through linear regression models [19]. An illustration of this is the work presented by [20], aiming to achieve torque performance using a convolutional neural network architecture, as also demonstrated by [21]. Similarly, ref. [22] employs a convolutional neural network to extract features from the distribution of the electric motor’s magnetic field to predict torque characteristics.

In the context of failure diagnostics, the data-driven approach has been demonstrated as suitable for various types of TIM applications, such as wind turbines [23]. When it comes to preventing failures, the data-driven approach relies on reading patterns from different types of sensors and classifying whether a failure exists or not. However, this is based on models that are able to perform complex linearizations in order to suggest the current behavior of the asset.

Furthermore, beyond the fault diagnostics employed in TIM, there are also many mechanical components mostly coupled on the TIM shaft that are important to assess, for instance pumps, conveyors, compressors, generators, and so on, as in the work by the authors [24], who have employed a data-driven architecture in order to detect failure on the gearbox.

The research applied to electric motors is continually advancing, and CNNs stand out as one of the most effective data-driven modeling strategies within the realm of deep learning. Utilizing CNNs in proposed approaches has demonstrated feasibility and practicality, yielding robust results across various scenarios related to electric motors. These applications span from motor fault detection [25] and analysis of specific components like bearings [26] to the assessment of motor performance for efficiency [27]. Furthermore, CNNs have proven valuable in the design of high-performance motors [28].

Nevertheless, despite the benefits of transforming time-series data into images for CNN-based analysis, it is crucial to take into account potential drawbacks. On the positive side, this approach allows for the extraction of spatial patterns and relationships, harnessing the powerful feature extraction capabilities of CNNs. Data augmentation techniques become more applicable, and compatibility with pre-trained models designed for image-related tasks offers a potential advantage. The visual nature of images enhances interpretability, aiding in exploratory data analysis. However, challenges include the potential loss of inherent temporal information present in time-series data, elevated computational demands associated with image processing, and the imperative need for meticulous parameter selection in the transformation process.

Therefore, the primary goal of this article is to introduce an innovative method for accurately estimating load torque without relying on intricate parameters or a detailed mathematical model of the TIM. This approach adopts a data-driven architecture that negates the necessity for deep TIM parameters. Instead, it utilizes a computational model founded on a supervised convolutional neural network tasked with learning and extracting features from a set of images generated from a periodic signal obtained from the TIM air gap.

The results demonstrated a good performance of this approach for load torque estimation using three different time windows, as well as 100 ms, 200 ms, and 400 ms from a magnetic flux, allowing us to assess the robustness of the image-based approach in industrial environments. When compared to other works in the literature, the main contributions of this work can be summarized as follows: (i) an approach capable of extracting features from images that reflect the magnetic flux and estimating load torque without the need for additional variables such as voltage, current or even speed; (ii) capability of estimating the load torque even with low-sized images created by very small time windows; (iii) this method can estimate load torque even in low load below 30% of the nominal load; and (iv) estimation error below 3.1% in a range spanning from 1.5% to 93.9% of the nominal load. Moreover, it should be mentioned that the image-based approach can be improved by using different approaches to processing the images; however, this research may help with rotor shaft speed-torque estimation under failure-in-service behavior, as well as the unbalanced power supply for induction motors applied to distinct operational conditions.

## 2. Magnetic Flux Signal Conversion into Images and the Convolutional Neural Network Model

### 2.1. Signal Processing for Image Construction

The digital representation of the continuous signal in the time domain originating from the Hall effect sensor is described through the function α:I⊂Z→R, or more succinctly as α:I→R, where *I* denotes a set of discrete points in the domain of integers Z, and R represents the corresponding amplitude value of the signal mapped onto the set of real numbers. This function, α(t), expresses the value of the continuous signal for every time instance *t* [29].

The sampling process involves determining the frequency at which the pulse train p(n)∈Z switches from 0 to 1 activation, set here in this work at 10 kHz. The sampling time $T_{s}$ of 0.1 ms signifies the activation time of p(n) between instances n∈I, representing how many times the magnetic flux analog signal is discretized within a time interval [30].

In this sense, the raw signal is discretized into 100,000 samples and stored in the indexed vector u[n]. Considering the raw signal spans 10 s with a sampling rate of 10 kHz, for each image creation, the vector u[n] is subdivided into smaller sets or windows W∈R. This subdivision aims to assess the load torque estimation using images created from periodic signals less than 1 s, for example, 100 ms, 200 ms, and 400 ms.

Therefore, to construct an image $\gamma_{i}^{j}\in R^{L\times C}$ with a time window $T_{w}$ of 200 ms, for instance, considering a sampling time $T_{s}$ of 0.1 ms, the interval $T_{c}$ between each window *W* from the vector u[n] spans 2000 samples. $T_{c}=\frac{T_{w}}{T_{s}}=\frac{200ms}{0.1ms}=2000\;\mathrm{samples}$. Notably, $T_{c}$ also represents the resolution of the image with dimensions of 40 × 50 (C×L) pixels.

In this case, $W_{i}\subset W=\left\{W_{1},W_{2},W_{3},\cdots ,W_{i}\right\}$ represents a smaller amount of samples, and $W_{\left(i,j\right)}\subset W_{i}=\left\{W_{\left(i,1\right)},W_{\left(i,2\right)},W_{\left(i,3\right)},\cdots ,W_{\left(i,j\right)}\right\}$ stands for each sample seen as a single pixel, while each image is created by the subsets association of *W*. Therefore, each subset $W_{i}\in R$ represents a row *L* for the image $\gamma_{i}^{j}$, where *i* denotes the *i*-th subset of *W* for L=50, and i∈{1,2,3,⋯,L}.

Thus, 1≤i≤L. Each subset $W_{i,j}\in R$ thus represents a sample of $W_{i}$, where *j* denotes the *j*-th index of a subset $W_{i,j}$. In the context of the image $\gamma_{i}^{j}$, *j* represents the columns of the image, where for C=40, j∈{1,2,3,⋯,C}; thereby, 1≤j≤C. It becomes possible to map the samples of the signal u[n] to the matrix $\gamma_{i}^{j}$ using the indices *i* and *j*, as expressed in Equation (1).

(1) $$u\left[\left(\left(i-1\right)\times C\right)+j\right]=W_{i,j}\to \gamma_{i}^{j}$$

Therefore, for each value of a real discrete sample between the minimum and maximum intervals, its corresponding representation in grayscale, ${⋃}_{k=0}^{k}Q\left[n\right]=u\left[n\right]$, for limits u[n]=−1 and u[n]=1, is determined using Equation (2).

(2) $$Q\left[n\right]=\mathrm{Round}\left(\frac{u[n]-\mathrm{MIN}(u)}{\mathrm{MAX}(u)-\mathrm{MIN}(u)}\right)\times k$$

where the transformation from real values to *R*-bit values in Z, 0≤R≤8, occurs, with the resolution of the integer value *k* representing the grayscale with the range 0≤k≤255, given that $k\in Z=2^{R-1}$.

Given that the time $T_{x}$ for each experiment is 10 s, it results in a total of 50 images. This relationship is expressed by the ratio $\frac{T_{x}}{T_{w}}=\frac{10s}{0.200s}=50\;\mathrm{images}$.

**Figure 1:**
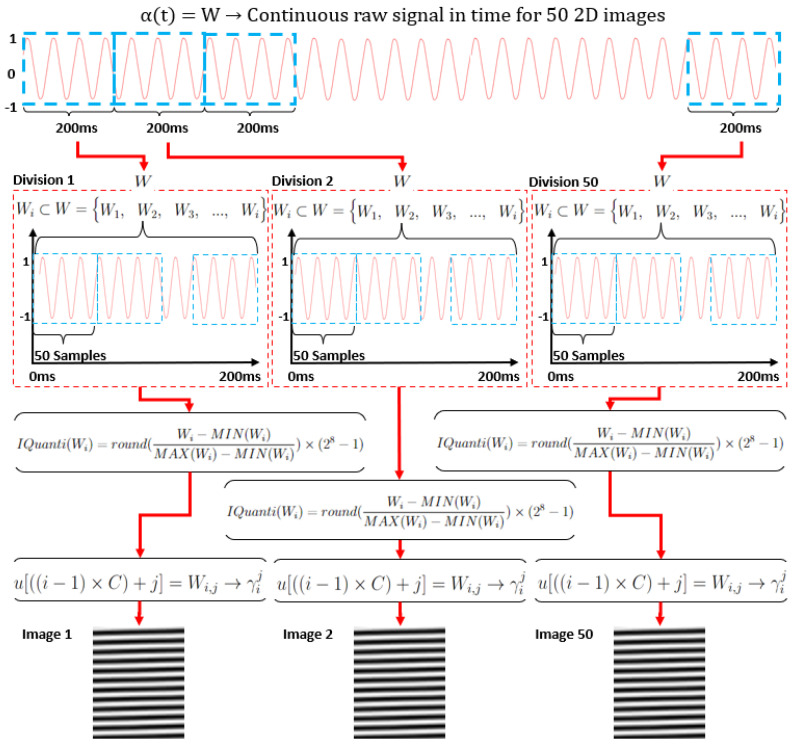
Image-based approach for time signal conversion.

Figure 1 presents a comprehensive illustration elucidating the stages involved in image processing until the creation of multiple images.

### 2.2. Convolutional Neural Network for Image-Based Regression

To estimate the load torque magnitude based on preprocessed images derived from the magnetic flux signal measured in the air gap of a TIM, the methodological process entailed investigating whether a supervised training convolutional neural network (CNN) model possessed the capability to estimate magnitudes from images created from temporal signals.

The present research has been carried out to explore various models and approaches within this domain. Different architectures such as VGG-16, LeNet-5, Inception V2, and V3, alongside some customized architectures, were tested. The primary focus was to understand how these architectures would perform on the regression task, considering variations in the sampling windows of the images. Through testing various CNNs, those with superior adaptation and regression capabilities were identified.

Table 1 showcases the hyperparameters and configurations for the investigated models that yielded better results, alongside model 1 to model 7 with the classical CNN architecture and the Inception model represented by model 8. The model’s definition was based on assigning different parameter combinations and evaluating the outcomes using the Adam, Adagrad, and RMSprop optimizers.

The configurations implemented in this work diverge from the standard settings of each version of the Inception CNN, such as versions 1, 2, 3, and 4. These versions were developed to address pattern classification problems, as presented in the works by the authors [31,32,33].

The Inception CNN model chosen in this work was modified, as detailed in Table 2. This approach was tailored to address the regression problem associated with estimating load torque in the TIM. To configure the network, the Keras framework was utilized, incorporating the essential functionalities of artificial neural networks.

Table 3 provides an overview of the architecture of the modified Inception CNN, detailing the convolutional layers and describing the number of channels, and the filter sizes used in each inception block, and their respective parallel convolutions.

These parameters embody the optimal architecture experimentally discovered for the research problem in this study. The interaction between the number of channels and the various filter sizes provides insight into the wide range of features that the model can integrate at each layer. This demonstrates the diversity of information extracted at different levels of abstraction.

## 3. Materials and Methods

### 3.1. Methodology for Load Torque Estimation

**Figure 2:**
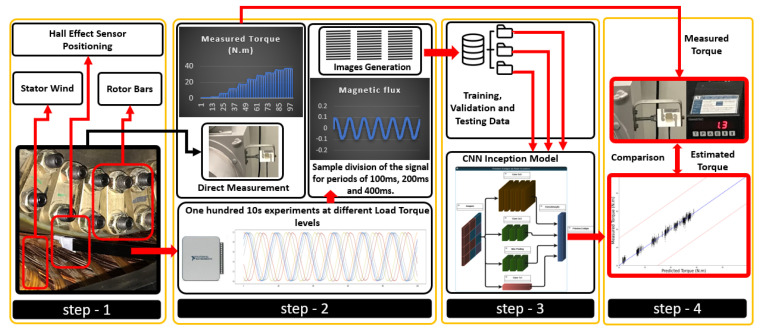
Methodology for torque estimation using the present approach.

The workflow of this study was split into phases or steps, as depicted in Figure 2.

In the first step, the Hall probe was installed inside the TIM to measure the magnetic flux density. The second step corresponds to the Hall sensor signal acquisition and signal division. Signal sampling fragmentation was conducted in each experiment, resulting in the creation of three distinct databases, one for a sampling period of 100 ms, another for a period of 200 ms, and a third one for a period of 400 ms. For instance, in the case of the 200 ms signal, the 100,000 samples were divided into 50 segments. This division was due to each segment containing 2000 samples, derived from $\frac{100,000}{2000}=50$. Similarly, for the 400 ms signal, the 100,000 samples were distributed across 25 segments, each consisting of 4000 samples, as calculated by $\frac{100,000}{4000}=25$. The same process is also performed for the 100 ms signal.

The signal preprocessing aims to generate images from the raw data collected within the motor air gap. This approach spatially allocates discrete values of each signal sample into an image grid. By doing so, the model can extract various patterns based on the applied load torque on the TIM. Consequently, after its training, it becomes capable of differentiating estimated torque load values corresponding to each image. This transformation into an image format enables the model to discern estimated load torque values concerning each processed image after its training. Essentially, the model learns the relationship between visual patterns within the generated images and the diverse levels of load torque applied to the motor. This ability allows the model to predict or differentiate these values based on the presented images.

In step 3, the generated images were stored for further CNN training and testing phases. Table 4 outlines the divisions and quantities of images for each of the three dimensions of images applied to the CNN Inception model. This setup allowed for an assessment of each image pattern across different hyperparameter combinations.

For instance, 10,000 images sized at 40 × 25 were distributed among training, validation, and test sets, with each group containing specific quantities of images. Similarly, the other two configurations, comprising images of 40 × 50 and 50 × 80, employed fewer images with variations in their respective dimensions and sampling times. Although larger-sized images accounted for a smaller portion of the dataset, the amount of information they contained remained constant. This was due to the number of samples staying the same, but distributed across different sample windows. This distribution facilitated the evaluation of how these parameters influenced model performance. In this context, a portion of the experiments was dedicated to training and validation, while the remainder was exclusively reserved for testing.

### 3.2. Experimental Apparatus

To be able to observe different levels of load torque, from the motor running at the no-load condition to its full load capacity, an electromagnetic brake known as a Foucault brake was used, coupled with a TIM. This brake, simply referred to as a Foucault current brake, operates due to Foucault currents or induced currents in the rotating disk installed on the TIM shaft. The brake structure is mechanically installed within the induction motor.

A conductive metal disk is mounted on the TIM shaft and is surrounded by a pair of stationary electromagnetic coils. When a continuous voltage level, for example, 20 V DC, is applied, a proportional level of electromagnetic field is generated around the disk. Consequently, the interaction between the opposing magnetic fields of the coils creates a resistance force against the movement of the disk installed on the TIM shaft.

The motor used in this study was a three-phase squirrel-cage induction motor, characterized by a rated power of 7.5 kW or 10 HP. This motor has 2 pairs of poles, 38 rotor bars, and a nominal synchronous speed of 1800 RPM. Additional information regarding the machine’s characteristics can be found in Table 5.

Similarly, applying a continuous voltage level, for example 240 V DC, yields an approximate load torque of 40 Nm as a consequence of the interaction between the brake system and the TIM during its nominal rotation. This voltage induces a proportional electromagnetic field, where this interaction allows the measurement, evaluation, and capture of the magnetic flux signal close to the TIM’s air gap. This signal analysis provides the dynamic in-service behavior of the TIM. During the experimental trials, the signal acquisition was performed by using a Hall effect probe, and its specifications are shown in Table 6.

The direct measurement of the load torque was accomplished using a load cell, designed to accurately capture and quantify torque exertion. This load cell was installed on the shaft of the TIM and integrated with a digital indicator to display the real-time measured torque values. These measurements are aligned with the specifications detailed in Table 7, enabling us to assess the torque applied in-service during the experimental observations.

## 4. Experimental Results

This section reveals the results obtained from each experimental test. The estimated torque in each scenario is divided into three distinct subsections, outlined as follows. The results obtained from investigating image preprocessing techniques in conjunction with the CNN Inception model are presented, alongside their respective behaviors concerning 54 combinations and applied hyperparameters. The study’s objectives focused on evaluating the predictive performance of the model after training, comparing it with direct load cell measurements. Additionally, the investigation delved into exploring the impact of different sample window dimensions derived from the magnetic flux signal.

Table 8 depicts a comprehensive set of hyperparameter combinations employed across the Adam, Adagrad, and RMSprop optimizers. These combinations encompass varying batch sizes (16, 32, and 64), training epochs (5 and 10), and diverse image dimensions (40 × 25, 40 × 50, and 50 × 80). This arrangement results in a total of 54 unique configurations, allowing for an extensive analysis of model performance encompassing the spectrum of 54 potential hyperparameter combinations.

During the training and cross-validation phase, the iterative process involves saving the model version with the lowest mean absolute error for each of the 10 stages. Consequently, this results in the availability of 10 distinct model versions. To ascertain the most effective performer among these variations, a step involves subjecting the test image dataset to each of these top 10 models. This evaluates and compares their individual capabilities to generalize beyond the training data.

The significance of this evaluation lies in determining the model’s ability to perform well on unseen data, ensuring it does not overfit or underfit. The process involves testing these 10 models against the test dataset, essentially assessing their adaptability to new, previously unseen inputs.

**Figure 3:**
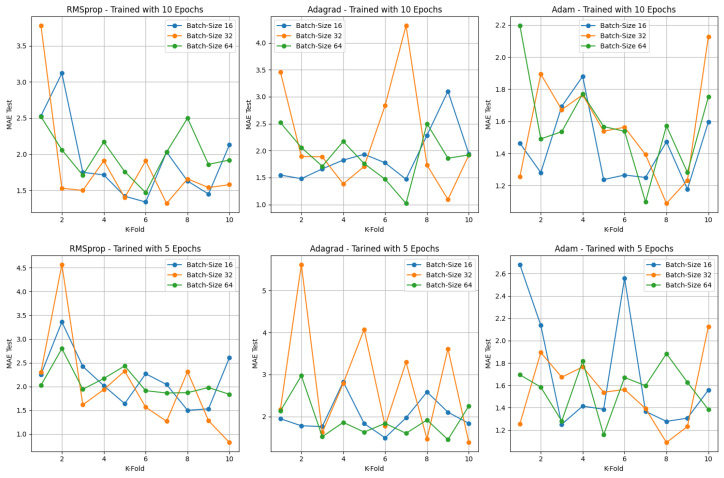
Results of 18 combinations of hyperparameters and optimizers for 5 and 10 training epochs.

As an example, in Figure 3, it is possible to observe a detailed representation of the performance exhibited by each optimizer, considering the image dimension of 40 × 50 pixels. This analysis considers their corresponding batch sizes across 5 and 10 training epochs for each *k*-fold iteration. Here, *k* represents each specific iteration within the 10-fold cross-validation framework, enabling an intricate examination of how these models perform across different settings and folds. This thorough exploration helps in discerning patterns of performance and robustness across varying hyperparameters and training iterations. This evaluation has been carried out for the other two image dimensions as well. The next subsections shows the torque estimation using each image dimension for each hyperparameter configuration.

### 4.1. Estimated Load Torque for Image Dimension of 40 × 50 Pixels

Within this context, Table 9 shows the outcomes obtained from 18 diverse combinations. These specific combinations are associated with images sized at 40 × 50 pixels, generated utilizing a sampling window of Ts=200 ms. The results encompass evaluations across training, validation, and test datasets.

**Figure 4:**
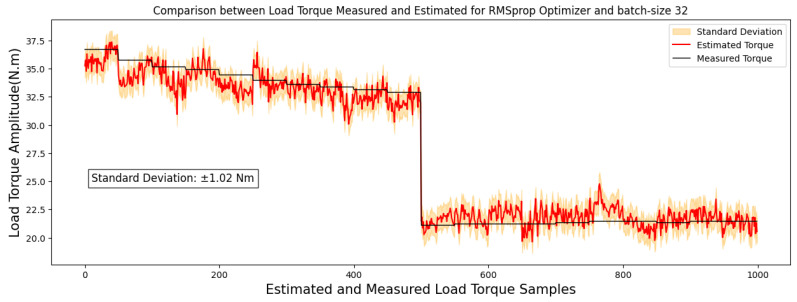
Correlation analysis results for images of 40 × 50 pixels.

**Figure 5:**
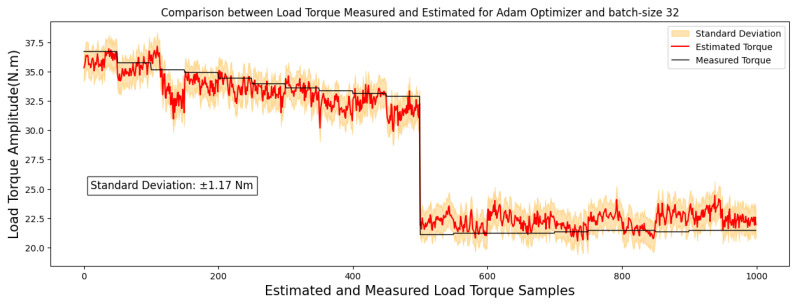
Correlation analysis results for images of 40 × 50 pixels.

Figure 4 and Figure 5 showcase comparisons based on the optimal outcomes obtained by the RMSprop and Adam optimizers applied to the test image set. They aim to illustrate the relationship between the measured and estimated TIM load torque.

Within the 40 × 50-pixel image standard, employing the Adam, Adagrad, and RMSprop optimizers alongside three distinct batch sizes and training cycles of 5 and 10 epochs, a distinct trend surfaced. Notably, the Adagrad optimizer, with a batch size of 64, showed superior performance based on the MAE and MAPE metrics across the test image set for the 10-epoch training cycle. Similarly, RMSprop displayed the most favorable outcome for the 5-epoch training cycle.

This observation underscores an intriguing distinction in optimizer behavior. Adagrad showcased an inclination towards improved performance with larger batch sizes. Conversely, both Adam and RMSprop demonstrated their best performance with a batch size of 32. However, it is worth noting that the difference in performance of the Adam optimizer for the 10-epoch between batch sizes 32 and 64 was not significantly discernible.

Moreover, a noteworthy observation emerges from the performance analysis of the Adam and RMSprop optimizers, indicating an enhancement in their performance with a reduction in the number of epochs. These findings suggest that, among the various batch sizes tested, the batch size of 32 consistently yielded optimal results. Notably, the reduction in training epochs to 5 yielded a reduction in error rates for both optimizers.

Specifically, upon reducing the training epochs, the RMSprop optimizer achieved a significantly lowered MAE of 0.82 Nm, showcasing an improvement in predictive accuracy. Similarly, the Adam optimizer, with a reduced training period, attained an MAE of 0.96 Nm, indicating an improvement in its predictive precision compared to higher epoch counts.

**Figure 6:**
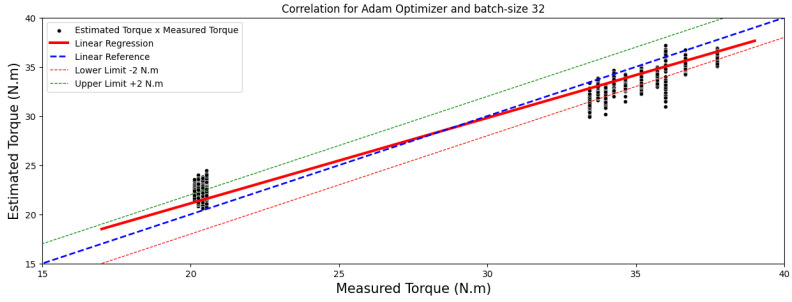
A comparison between estimated and measured load torque for 40 × 50-pixel image.

**Figure 7:**
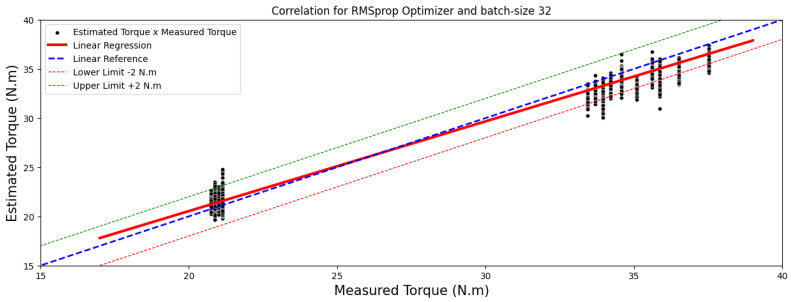
A comparison between estimated and measured load torque for 40 × 50-pixel image.

Moreover, the top-performing models, identified by a MAE below 1 Nm, are illustrated in images sized at 40 × 50 pixels, highlighted in Figure 6 and Figure 7. These regressions distinctly display a linear correlation among the observed outcomes within each model. This correlation emphasizes that variations in the measured torque variable have a proportional impact on the estimated torque variable.

### 4.2. Estimated Load Torque for Image Dimension of 40 × 25 Pixels

Similarly, the 40 × 25-pixel images created using a sampling window of Ts=100 ms were generated from a thousand samples, constituting the image set with the smallest sampling window, as indicated in Table 10. This set comprised a total of 10,000 images. These images underwent evaluation with the CNN Inception model, generating outcomes for a total of 18 configurations across both 5- and 10-epoch training settings.

During the 5-epoch training phase, the Adam optimizer displayed an improvement in performance as the batch size increased from 16 to 32. However, when tested with a batch size of 64, there was a rise in the test MAE, indicating a potential saturation or limitation of the model. This optimizer yielded the best result among these 5-epoch hyperparameter combinations. On the contrary, the RMSprop optimizer did not exhibit a clear trend in performance with regard to batch size variation. The results displayed fluctuations without a distinct trajectory towards either improved or deteriorating performance as the batch size increased.

In the case of Adagrad, a reduction in test MAE was observed as the batch size increased from 16 to 32, signifying an enhancement in model performance. However, testing with a batch size of 64 led to an increase in test MAE, suggesting a performance decline compared to the 32 batch size. Considering the 10-epoch training phase, the only prominent result was the RMSprop optimizer, with an MAE value of 0.97 Nm.

**Figure 8:**
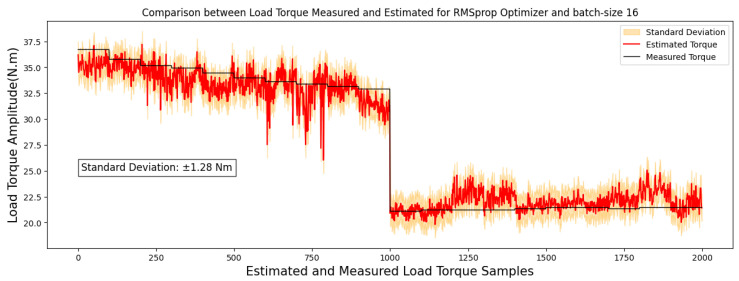
Correlations analysis for 40 × 25-pixel image results.

**Figure 9:**
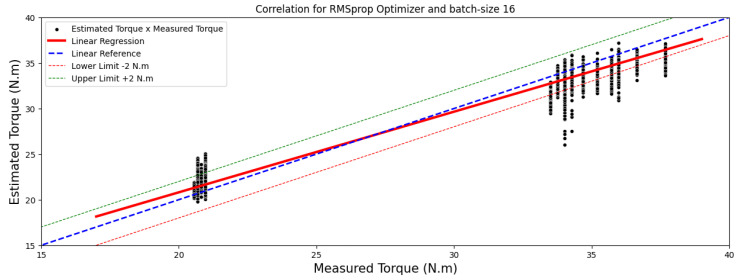
A comparison between estimated and measured load torque for 40 × 25-pixel image.

Comparison between the results from Table 9 and Table 10 suggests that while the 40 × 25-pixel image format demonstrated good performance, the model exhibited superior generalization with 40 × 50-pixel images. Based on the outcomes presented in Table 10, for 40 × 25-pixel images, the configuration that exhibited the best performance, achieving the lowest mean absolute error for the test set, was the model utilizing the RMSprop optimizer, trained over 10 epochs with a batch size of 16, and its estimation compared with real load torque is outlined in Figure 8 and Figure 9.

### 4.3. Estimated Load Torque for Image Dimension of 50 × 80 Pixels

The 50 × 80-pixel images, sampled with a window interval of Ts set at 400 ms, constitute the dataset with the largest sampling window, as indicated in Table 4. This set comprises a total of 2500 images, which were processed through the CNN Inception model, resulting in evaluations for 18 combinations involving 5- and 10-epoch training, as depicted in Table 11.

Regarding the outcomes illustrated in Table 11, the mean absolute error values for the test set indicate a noticeable trend concerning the Adam optimizer. There was a reduction in error as the batch size increased from 16 to 32. However, a slight decline in performance was observed when using a batch size of 64, making Adam the optimal optimizer among the 10-epoch hyperparameter combinations. Adagrad showcased a consistent improvement pattern in performance with increasing batch sizes. Conversely, RMSprop exhibited a more stable behavior, maintaining similar error values across different batch sizes. In the outcomes for the 5-epoch training, the Adam optimizer exhibited a decrease in performance as the batch size increased. Conversely, Adagrad showcased a tendency to reduce the MAE with larger batch sizes. Regarding RMSprop, its optimal performance was observed with a batch size of 32.

Comparison between Table 9 and Table 10 highlights a discernible trend, quite similar to the 40 × 50-pixel images, in which the model’s performance notably improved when the training epochs were reduced from 10 to 5 for the 50 × 80-pixel images. This observation suggests a potential pattern where larger image dimensions could potentially yield better outcomes with a reduced number of training epochs. However, confirming this correlation definitively necessitates further in-depth experimentation under these specific conditions.

**Figure 10:**
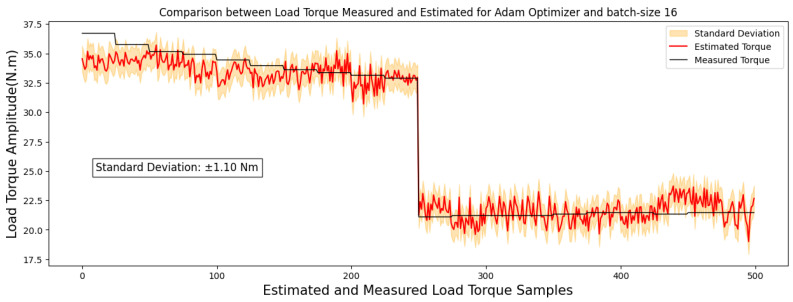
Correlations analysis for 50 × 80-pixel image results.

The significant understanding gleaned from Table 11 is visually depicted in Figure 10, effectively showcasing the comparative analysis between the actual measured torque and the torque estimated by the model. This visualization distinctly underscores the model’s ability to generalize, particularly when utilizing the Adam optimizer alongside a batch size of 16 for 50 × 80-pixel images.

Therefore, it is essential to emphasize that out of the 54 hyperparameter combinations analyzed, training with 5 epochs consistently yielded superior results. This finding implies that while achieving satisfactory outcomes with 10 epochs, the hyperparameter configuration of 5 epochs appears to be the most effective under the tested conditions and specifically for the problem addressed in this work.

**Figure 11:**
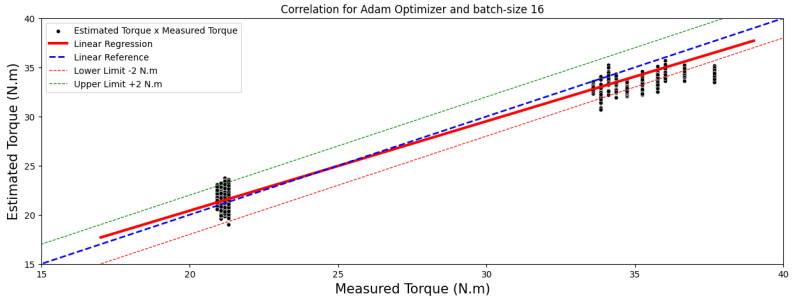
A comparison between estimated and measured load torque for 50 × 80-pixel image.

To assess the correlations between the measured torque and estimated torque, the results are depicted graphically using regressions, as illustrated for images sized at 40 × 25 pixels in Figure 9 and for images sized at 50 × 80 pixels in Figure 11.

Assessing the correlation levels between measured and estimated load torque across the top four models, calculations for both Pearson and Spearman correlation coefficients were conducted. The comprehensive findings are summarized in Table 12.

Based on the data in Table 12, the Pearson correlation coefficients reveal strong linear relationships, scoring 0.99 and 0.98 for RMSprop and 0.99 for Adam optimizer. Conversely, Spearman coefficients, registering at 0.85 and 0.86, suggest a robust but not necessarily linear correlation between the variables.

Based on these findings, it is evident that the most efficient training outcomes were achieved within 5 epochs. Across the three image dimensions, the Adam, Adagrad, and RMSprop optimizers demonstrated good results in both training and validation. Noteworthy is RMSprop’s best performance, in two of the top four results, particularly with the 200 ms sample window image pattern. The ideal image size for this study appears to be 40 × 50 pixels, with RMSprop as the preferred optimizer, showcasing superior performance in two instances with a batch size of 32, as detailed in Table 13.

## 5. Conclusions

In conclusion, several approaches suggest low-error load torque estimation, particularly when operating close to or above 30% of the nominal load. However, according to the authors of [2], estimating torque accurately at low loads, especially below 30% of the TIM’s nominal load, remains a challenge. This study delves into the model’s training and generalization capabilities specifically at low load levels, evaluating torque estimation below 30% of the TIM’s nominal load, thus examining estimations below 12 Nm. For instance, in the approach by [14], torque estimation showed less than 2% error for all points above 30% of the nominal load and less than 4% error below that threshold. Similarly, the authors of [34] emphasize their approach’s high precision in torque estimation with load intervals of 25% to 100%.

Additionally, in the work by [35], a maximum error of around 4.03% was reported. However, as highlighted by the authors, low slip condition could compromise their model’s performance. This contextualization is important for understanding the robustness and capability of the employed method, showing its ability to generalize results post-supervised-training, maintaining a mean absolute percentage error below 3.1% across the entire load range, spanning from 1.5% to 93.9% of the TIM’s nominal load. It is worth emphasizing the significance of the covered mechanical power range, particularly in areas deemed low load in the literature, where operational conditions resemble no-load operation. These conditions encompass operational behavior without load, representing 1.5% of the TIM’s nominal load, equivalent to 0.59 Nm, up to 29.8% of the nominal load, which equals 11.91 Nm. This characteristic underscores the model’s ability to handle low torque intensity scenarios up to conditions nearing its maximum capacity.

When compared to other studies, the main contributions of this research can be summarized as follows: (i) approach capable of estimating load torque even for 1.5% of the nominal load; (ii) a computational model capable of generalizing low-sized images even with just 5 and 10 training epochs; and (iii) an approach capable of estimating load torque without needing other features than magnetic flux. Although this study presents a novel approach for estimating load torque in TIM, yielding good results, future research avenues could enhance its applicability by applying this proposed methodology considering the TIM running under failure conditions, such as broken rotor bars [36], shaft imbalance [37], and angular misalignment [38]. Furthermore, investigations regarding the performance of the model under various ranges of power supply frequency, for instance spanning from 0 to 60 Hz, could be conducted. This could expand the applicability of this approach. Additionally, further studies could delve into alternative image processing methods, such as Gramian angular field or morphological mathematics, to expand the methodology’s scope and potentially optimize its efficacy in load torque estimation.

## Figures and Tables

**Table 1:** Comparison of architectures and hyperparameters among the evaluated CNN models.

Model	Conv2D	Momentum	Dense Layer	Filters	Channels
1	5	0.9	256, 1	(11, 11), (7, 7)	16, 32, and 64
2	3	0.6	256, 1	(3, 3), (5, 5)	16 and 32
3	7	0.9	1024, 1	(5, 5), (7, 7)	64 and 128
4	4	0.9	512, 256, 1	(11, 11)	16 and 32
5	2	0.8	100, 1	(3, 3)	16 and 32
6	5	0.9	256, 256, 1	(5, 5)	16 and 32
7	8	0.2, 0.8	110, 1	(7, 7), (11, 11)	16 and 32
8	16	0.5	320, 50, 1	Table 2	Table 2

**Table 2:** Details of the hyperparameters and architecture of the modified Inception CNN model.

Hyperparameter	Value
Conv2D Layers	16
Filters	Refer to Table 3
Filter Sizes	Refer to Table 3
Activation	ReLU (in all convolutions)
Batch Normalization	Present in all convolutions
Pooling Layers	4 MaxPooling2D
Dense Layers	3
Neurons in Dense Layers	320, 50, 1
Activation in Dense Layers	Linear
Dropout	None

**Table 3:** Details of hyperparameters for the modified Inception CNN model.

Layer	Kernel	Filter Sizes
Conv_1	32	(1, 1)
Conv_2	32	(1, 1), (3, 3), (7, 7)
Conv_3	32	(1, 1), (1, 7), (7, 1), (1, 11), (11, 1)
Conv_4	32	(1, 1)
Conv_5	32	(1, 1), (1, 7), (7, 1), (1, 11), (11, 1)
Conv_6	32	(1, 1), (1, 7), (7, 1), (1, 11), (11, 1)
Conv_7	48	(1, 1)
Conv_8	48	(1, 1), (1, 3), (3, 1)
Conv_9	48	(1, 1), (3, 3), (7, 1), (1, 7)
Conv_10	64	(1, 1)
Conv_11	64	(1, 1), (1, 5), (5, 1)
Conv_12	64	(1, 1), (5, 5), (9, 1), (1, 9)
Conv_14	128	(1, 1)
Conv_15	128	(1, 1), (1, 7), (7, 1), (11, 11), (11, 1), (1, 11)
Conv_16	128	(1, 1), (11, 11), (11, 1), (1, 11)

**Table 4:** Description of division and quantity for three image patterns.

Image Dimensions	Image Quantity	Train	Validation	Test
40 × 25	10,000	7200	800	2000
40 × 50	5000	4500	500	1000
50 × 80	2500	2250	250	500

**Table 5:** Motor specifications.

Motor Parameter	Value
Rated power	7.5 kW
Rated torque	41 Nm
Rated speed and slip	1740 r/min and 3.4%
Stator slot count	48
Number of pole pairs	2
Rotor bar count	38
Nominal frequency	60 Hz
Nominal voltage	220 V
Air gap distance	0.8 mm

**Table 6:** Hall sensor specifications.

Parameters	Range Classification
Maximum output	2.0 V
Conversion	1 T/V or 0.1 T/V
Precision	2% at 25 °C
Frequency response	DC to 5 kHz (1 T/V) and 700 Hz (0.1 T/V)
Sensor type	Transverse Hall

**Table 7:** Load cell and display specifications.

Load Cell Specifications	Indicator Specifications
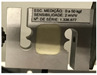	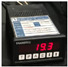
Sensor sensitivity = 2 mV/V	Display = 3 ½ digits (LED)
Nominal scale = 0 to 50 kgf	Nominal voltage = 110/220 V AC
Input impedance = 400 ± 15 Ω	Frequency = 60 Hz
Thermal response = −5 °C to +60 °C	Thermal response = 0 °C to +50 °C
Sensor type = Wheatstone bridge	Analog output = 0 to 10 V DC

**Table 8:** Hyperparameter combinations for different optimizers and image dimensions.

Optimizer Options	Batch Size Options	Epoch Options	Image Dimension Options
Adam	16, 32, 64	5, 10	40 × 25, 40 × 50, 50 × 80
Adagrad	16, 32, 64	5, 10	40 × 25, 40 × 50, 50 × 80
RMSprop	16, 32, 64	5, 10	40 × 25, 40 × 50, 50 × 80

**Table 9:** Images of 40 × 50 pixels—18 experimental results for 5 and 10 different epochs.

Setup			Test		Attributes	
Optimizer	Batch	Epochs	MAE	MAPE	*Fs*	*Ts*
Adam	16	5	1.25	5.1%	10 kHz	200 ms
Adagrad	16	5	1.49	6.1%	10 kHz	200 ms
RMSprop	16	5	1.5	5%	10 kHz	200 ms
Adam	32	5	0.96	3.7%	10 kHz	200 ms
Adagrad	32	5	1.38	5.1%	10 kHz	200 ms
RMSprop	32	5	0.82	3%	10 kHz	200 ms
Adam	64	5	1.15	4.2%	10 kHz	200 ms
Adagrad	64	5	1.45	4.9%	10 kHz	200 ms
RMSprop	64	5	1.83	7.6%	10 kHz	200 ms
Adam	16	10	1.17	4.8%	10 kHz	200 ms
Adagrad	16	10	1.46	5.1%	10 kHz	200 ms
RMSprop	16	10	1.34	5.5%	10 kHz	200 ms
Adam	32	10	1.08	4.5%	10 kHz	200 ms
Adagrad	32	10	1.09	4.1%	10 kHz	200 ms
RMSprop	32	10	1.32	5.2%	10 kHz	200 ms
Adam	64	10	1.09	4%	10 kHz	200 ms
Adagrad	64	10	1.02	3.9%	10 kHz	200 ms
RMSprop	64	10	1.47	5.8%	10 kHz	200 ms

**Table 10:** Images of 40 × 25 pixels—18 experimental results for 5 and 10 different epochs.

Setup			Test		Attributes	
Optimizer	Batch	Epochs	MAE	MAPE	*Fs*	*Ts*
Adam	16	5	1.3	4.4%	10 kHz	100 ms
Adagrad	16	5	1.94	7.5%	10 kHz	100 ms
R msprop	16	5	1.16	3.9%	10 kHz	100 ms
Adam	32	5	1.07	3.6%	10 kHz	100 ms
Adagrad	32	5	1.1	4.4%	10 kHz	100 ms
R msprop	32	5	1.21	4.4%	10 kHz	100 ms
Adam	64	5	1.22	4.1%	10 kHz	100 ms
Adagrad	64	5	1.65	6%	10 kHz	100 ms
R msprop	64	5	1.09	3.9%	10 kHz	100 ms
Adam	16	10	1.59	5.6%	10 kHz	100 ms
Adagrad	16	10	1.47	5.3%	10 kHz	100 ms
R msprop	16	10	0.97	3.5%	10 kHz	100 ms
Adam	32	10	1.52	6.2%	10 kHz	100 ms
Adagrad	32	10	1.41	5.3%	10 kHz	100 ms
R msprop	32	10	1.48	5.9%	10 kHz	100 ms
Adam	64	10	1.29	4.6%	10 kHz	100 ms
Adagrad	64	10	1.59	5.8%	10 kHz	100 ms
R msprop	64	10	1.69	6.2%	10 kHz	100 ms

**Table 11:** Images of 50 × 80 pixels—18 experimental results for 5 and 10 different epochs.

Setup			Test		Attributes	
Optimizer	Batch	Epochs	MAE	MAPE	*Fs*	*Ts*
Adam	16	5	0.91	3.3%	10 kHz	400 ms
Adagrad	16	5	1.71	6.8%	10 kHz	400 ms
RMSprop	16	5	1.45	6%	10 kHz	400 ms
Adam	32	5	1.14	4.3%	10 kHz	400 ms
Adagrad	32	5	1.58	6.1%	10 kHz	400 ms
RMSprop	32	5	1.4	5.2%	10 kHz	400 ms
Adam	64	5	1.21	4.6%	10 kHz	400 ms
Adagrad	64	5	1.38	4.9%	10 kHz	400 ms
RMSprop	64	5	1.56	6%	10 kHz	400 ms
Adam	16	10	1.31	5.3%	10 kHz	400 ms
Adagrad	16	10	1.63	6.3%	10 kHz	400 ms
RMSprop	16	10	1.34	5.1%	10 kHz	400 ms
Adam	32	10	1.02	4%	10 kHz	400 ms
Adagrad	32	10	1.6	6.5%	10 kHz	400 ms
RMSprop	32	10	1.27	4.6%	10 kHz	400 ms
Adam	64	10	1.06	4.1%	10 kHz	400 ms
Adagrad	64	10	1.27	4.8%	10 kHz	400 ms
RMSprop	64	10	1.21	4.5%	10 kHz	400 ms

**Table 12:** Correlation results for optimization methods.

Correlation Analysis among the Top 4 Models			
MAE Test	Optimizer	Pearson	Spearman
0.82 (Table 9)	RMSprop	0.99	0.85
0.96 (Table 9)	Adam	0.99	0.86
0.97 (Table 10)	RMSprop	0.98	0.86
0.91 (Table 11)	Adam	0.99	0.85

**Table 13:** Images of 40 × 50 pixels—results of experiments with different optimizers and hyperparameters.

Configuration			Test		Attributes	
Optimizer	Batch	Epochs	MAE	MAPE	*Fs*	*Ts*
RMSprop	32	5	0.82	3%	10 kHz	200 ms

## Data Availability

Data are contained within the article.
